# Effects of C-peptide on Microvascular Blood Flow and Blood Hemorheology

**DOI:** 10.1080/15438600490424532

**Published:** 2004

**Authors:** T. Forst, T. Kunt

**Affiliations:** 1 Institute for Clinical Research and Development Mainz D-55130 Germany; 2 Department of Internal Medicine ZMH Abu Dhabi United Arab Emirates

## Abstract

Beside functional and structural changes in vascular biology, alterations in the rheologic properties of blood cells mainly determines to an impaired microvascular blood flow in patients suffering from diabetes mellitus. Recent investigations provide increasing evidence that impaired C-peptide secretion in type 1 diabetic patients might contribute to the development of microvascular complications. C-peptide has been shown to stimulate endothelial NO secretion by activation of the Ca^2+^ calmodolin regulated enzyme eNOS. NO himself has the potency to increase cGMP levels in smooth muscle cells and to activate Na^+^ K^+^ ATPase activity and therefore evolves numerous effects in microvascular regulation. In type 1 diabetic patients, supplementation of C-peptide was shown to improve endothelium dependent vasodilatation in an NO-dependent pathway in different vascular compartments. In addition, it could be shown that C-peptide administration in type 1 diabetic patients, results in a redistribution of skin blood flow by increasing nutritive capillary blood flow in favour to subpapillary blood flow. Impaired Na^+^ K^+^ ATPase in another feature of diabetes mellitus in many cell types and is believed to be a pivotal regulator of various cell functions. C-peptide supplementation has been shown to restore Na^+^ K^+^ATPase activity in different cell types during in vitro and in vivo investigations. In type 1 diabetic patients, C-peptide supplementation was shown to increase erythrocyte Na^+^ K^+^ATPase activity by about 100%. There was found a linear relationship between plasma C-peptide levels and erythrocyte Na^+^ K^+^ATPase activity. In small capillaries, microvascular blood flow is increasingly determined by the rheologic properties of erythrocytes. Using laser-diffractoscopie a huge improvement in erythrocyte deformability could be observed after C-peptide administration in erythrocytes of type 1 diabetic patients. Inhibition of the Na^+^ K^+^ATPase by Obain completely abolished the effect of C-peptide on erythrocyte deformability. In conclusion, C-peptide improves microvascular function and blood flow in type 1 diabetic patients by interfering with vascular and rheological components of microvascular blood flow.


*Experimental Diab. Res.*, 5:51–64, 2004
Copyright © Taylor and Francis Inc.
ISSN: 1543-8600 print / 1543-8619 online
DOI: 10.1080/15438600490424532

Taylor & Francis
Taylor & Francis Group

### Effects of C-peptide on Microvascular Blood Flow and Blood Hemorheology

**T. Forst[1] and T. Kunt[2]**

[1]*Institute for Clinical Research and Development, Mainz, Germany*
[2]*Department of Internal Medicine, ZMH, Abu Dhabi, United Arab Emirate*

**Beside functional and structural changes in vascular biology, alterations in the rheologic properties of blood cells mainly determines to an impaired microvascular blood flow in patients suffering from diabetes mellitus. Recent investigations provide increasing evidence that impaired C-peptide secretion in type 1 diabetic patients might contribute to the development of microvascular complications. C-peptide has been shown to stimulate endothelial NO secretion by activation of the Ca2$^{+}$ calmodolin regulated enzyme eNOS. NO himself has the potency to increase cGMP levels in smooth muscle cells and to activate Na$^{+}$K$^{+}$ATPase activity and therefore evolves numerous effects in microvascular regulation. In type 1 diabetic patients, supplementation of C-peptide was shown to improve endothelium dependent vasodilatation in an NO-dependent pathway in different vascular compartments. In addition, it could be shown that C-peptide administration in type 1 diabetic patients, results in a redistribution of skin blood flow by increasing nutritive capillary blood flow in favour to subpapillary blood flow. Impaired Na$^{+}$K$^{+}$ATPase in another feature of diabetes mellitus in many cell types and is believed to be a pivotal regulator of various cell functions. C-peptide supplementation has been shown to restore Na$^{+}$K$^{+}$ATPase activity in different cell types during in vitro and in vivo investigations. In type 1 diabetic patients, C-peptide supplementation was shown to increase erythrocyte Na$^{+}$K$^{+}$ATPase activity by about 100%. There was found a linear relationship between plasma C-peptide levels and erythrocyte Na$^{+}$K$^{+}$ATPase activity. In small capillaries, microvascular blood flow is increasingly determined by the rheologic properties of erythrocytes. Using laser-diffractoscopie a huge improvement in erythrocyte deformability could be observed after C-peptide administration in erythrocytes of type 1 diabetic patients. Inhibition of the Na$^{+}$K$^{+}$ATPase by Obain completely abolished the effect of C-peptide on erythrocyte deformability. In conclusion, C-peptide improves microvascular function and blood flow in type 1 diabetic patients by interfering with vascular and rheological components of microvascular blood flow.**

**Keywords** C-peptide; Microvascular Blood Flow; Nitric Oxide; Sodium-Potassium-Pump; Type 1 Diabetes

### INTRODUCTION

### Pathophysiology of Vascular Complications in Diabetes Mellitus

Since the discovery of insulin in 1922 by Banting and Best, the most frequent complications of type 1 diabetes mellitus have changed from acute metabolic disturbances to the long-term complications retinopathy, nephropathy, and neuropathy. Although hyperglycemia is recognized as an important pathogenetic factor in the development of these diabetic complications, the precise mechanisms whereby diabetes precipitates these complications, are still largely unknown. Furthermore, it is well-known also that in type 1 diabetic patients with good metabolic control, microvascular complications cannot be sufficiently prevented. Therefore, the possibility may be considered that other factors than the lack of insulin secretion or the elevated blood glucose levels might be involved in the pathogenesis of diabetic complications.

Numerous functional and structural microvascular disturbances have been reported for patients suffering from type 1 diabetes. These microvascular disturbaces are thought to be

Received 12 March 2003; accepted 2 October 2003.
Address correspondence to Thomas Forst, Institute for Clinical Research and Development, Mainz D-55130, Germany. E-mail: thomasf@ikfe.de implicated in the development of diabetic complications, such as retinopathy, neuropathy, and nephropathy, and the development of foot skin ulceration. The microcirculation is concerned with the transport and exchange of nutrients and waste products of metabolism, tissue defense and repair, and the often conflicting demand of maintenance tissue fluid economy. Early type 1 diabetes is characterized by increased microvascular blood flow, increased shear stress, and tangential pressure on microvascular endothelium. In consequence, microvascular sclerosis occurs, involving basement membrane thickening as well as arteriolar hyalinosis, which limits the capacity of the microvasculature to dilate at times of increased demand (Tooke, 1999). The etiology of diabetic microvascular dysfunction is not fully understood, but several mechanisms that appear to act synergistically as pathogenetic factors in the setting of microvascular blood flow regulation are discussed. In addition to functional abnormalities of the microvasculature, such as disturbed neurovascular responses (Forst et al., 1997; Netten et al., 1996), alterations in endothelial function (Johnstone et al., 1993), and an increased intracapillary pressure (Sandemann et al., 1992), several hemorheological disturbances have been described in patients with diabetes mellitus. An increase in leucocyte-endothelial interactions (Kunt et al., 1998a), increased blood viscosity (Ernst and Matrai, 1986; Barnes et al., 1977), and changes in the rheologic properties of red blood cells (Finotti and Palatini, 1986; McMillan et al., 1998) may contribute to the observed microvascular alterations in blood flow. These early microvascular and hemorheological abnormalities may precede structural vascular changes, which occur later in the course of the microvascular disease (Rayman et al., 1995).

### Pathophysiology of Vascular Endothelium in Diabetes Mellitus

A hypothesis of the initial event of microvascular disease is endothelial dysfunction, defined pragmatically as changes in the concentration of the chemical messangers produced by the endothelial cell. (Calles-Escandon and Cipolla, 2001). The endothelial cell lines the internal lumen of the vasculature and serves as an interface between circulating blood and vascular smooth muscle cell. In addition to serving as a physical barrier between the blood and tissues, the endothelial cell facilitates a complex array of functions in intimate interaction with the vascular smooth muscle cell, as well as cells within the blood stream. There are several substances released from the endothelial cells under physiologic circumstances that modify the vascular tone, e.g., nitric oxide (NO), prostaglandins, and many other peptides, such as thrombin, substance P, bradykinin, serotonin, etc. (Colwell and Lopes-Virella, 1988).

The most important finding concerning the physiology of vascular tone was the discovery of the so-called endothelium derived relaxing factors (EDRF) by Robert Furchgott and coworkers in 1980 (Furchgott and Zawadzki, 1980). They were able to demonstrate that rings of isolated rabbit aorta contracted with phenylephrine failed to relax after administration of acetylcholine if the intimal layer, i.e., the endothelium, was removed from the blood vessel mechanically prior to the investigation. Moreover, NO was identified as the primary vasodilator released from the endothelium (Palmer et al., 1987). Endogeneous NO is produced within the endothelial cell through the conversion of the amino acid L-arginine to L-citrulline by the enzyme endothelial NO synthase (eNOS), one of several isoforms of the enzyme. eNOS is regulated by $Ca^{2+}$-calmodulin with NADPH. NO produced by the endothelial cell diffuses into the blood stream and to the vascular smooth muscle cell. In the vascular smooth muscle cell, it activates the enzyme guanylate cyclase, leading to an increase in cyclic GMP with subsequent vasorelaxation (Figure 1).

Many agonists, such as acetylcholine, are able to initiate the release of NO by increasing the endothelial cell calcium (Danthuluri et al., 1988). This can be mediated by receptor activation of phospholipase C, with the subsequent formation of inositol triphosphate and release of intracellular calcium stores, and by enhancing the influx of extracellular calcium as well; e.g., in an experimental model, the ionophore A23187 is able to increase the level of intracellular calcium without involving endothelial receptors (Peach et al., 1987).

Studies in the field of diabetology have always been focusing especially on the effect of hyperglycemia on vascular dysfunction. There is evidence that the abnormality of the endothelium in diabetes mellitus results from abnormal production of vasoconstrictor prostaglandins because this dysfunction could be normalized by cyclooxygenase inhibitors or by inhibition of thromboxane $A_2$/prostaglandin $H_2$ receptors (Tesfamariam et al., 1989).

Early studies suggested that hyperglycemia may contribute to vascular dysfunction by enhanced synthesis of sorbitol via the aldose reductase pathway, causing depletion of the intracellular pool of *myo*-inositol (Greene et al., 1987). Inhibition of aldose reductase as well as addition of *myo*-inositol in an aortic ring model resulted in a normal endothelium-dependent relaxation in response to acetylcholine. It is tempting to speculate that this depletion of *myo*-inositol decreases the turnover of phosphatidylinositol, which is involved in the regulation of $Na^+$,$K^+$-ATPase.

$Na^+$,$K^+$ATPase activity has been found to be attenuated in various cell types under diabetic conditions (Wald et al., 1993; Gerbi et al., 1997; Vague et al., 1997). It has also been shown that hyperglycemia inhibits $Na^+$,$K^+$-ATPase activity by

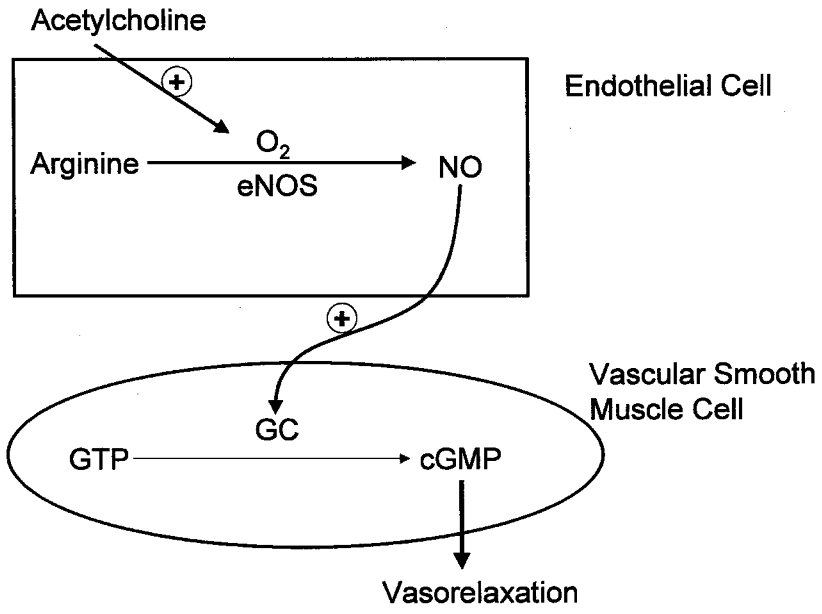

**FIGURE 1**
Mechanism of endothelial nitric oxide synthesis and subsequent vasorelaxation.

an endothelium dependent pathway (Simmons et al., 1986). $Na^+$,$K^+$-ATPase is a plasma membrane–associated protein complex that is expressed in most eukaryotic cells. It couples the energy released in the intracellular hydrolysis of ATP to the transport of cellular ions, a major pathway for the controlled translocation of sodium and potassium ions across the cell membrane. Therefore, $Na^+$,$K^+$-ATPase is able to control directly or indirectly many essential cellular functions, e.g., cell volume, free calcium concentrations, and membrane potential (Rose and Valdes, Jr., 1994). Although there are tissue differences and tissue-specific regulations of $Na^+$,$K^+$-ATPase activity, hyperglycemia and diabetes are predominantly characterized by a decrease of ouabain-sensitive $Na^+$,$K^+$-ATPase activity, leading to disturbances of main cellular mechanisms (see above), e.g., to an increased intracellular calcium level and to increased vascular contractility, both of which promoting vascular complications in diabetes mellitus.

$Na^+$,$K^+$ATPase activity is involved in vascular modulation based on a complex interaction between $Na^+$,$K^+$ pump activity and an endothelium-dependent increase of NO (Rapoport et al., 1985; Tack et al., 1996). On the other hand, NO and cyclic GMP have been shown to increase vascular $Na^+$,$K^+$ATPase activity, with subsequent vasorelaxation (Gupta et al., 1994; Rand and Garland, 1992).

The impairment of $G_i$-proteins also seems to be a common mechanism of vascular dysfunction in diabetes mellitus; e.g., $G_i$-protein is deficient in streptozotocin-induced diabetic rats (Gawler et al., 1987). Pertussis toxin, which is able to inactivate $G_i$-protein by ADP ribosylation, inhibits the vascular relaxation caused by acetylcholine. Actually, the impairment of vascular relaxation due to pertussis toxin appears to be very similar to the vascular dysfunction caused by hyperglycemia itself, suggesting the involvement of $G_i$-proteins in this mechanism.

Protein kinase C (PKC) activation is another highly potential candidate explaining the vascular dysfunction in hyperglycemia (Lee et al., 1989). King and colleagues were able to demonstrate that phorbol myristate acetate (PMA) exerts a similar effect on endothelial cell function as seen after exposure to high concentrations of glucose. For example, PMA was able to increase the acetylcholine-induced release of vasoconstrictor prostaglandins in isolated rabbit aortic rings. Moreover, this increased release of vasoconstricor prostaglandins was prevented by addition of PKC inhibitors. The activation of PKC, in turn, inactivates $G_i$-protein by phosphorylation. Hence, it is very likely that this mechanism may be responsible for the vascular dysfunction caused by hyperglycemia in diabetes mellitus.

In summary, there is increasing evidence concerning the underlying mechanisms of vascular dysfunction in diabetes mellitus, especially under hyperglycemic conditions. In contrast, there has been almost no evidence so far that explains vascular dysfunction in diabetes mellitus in the absence of hyperglycemia. This is the field of research, predominantly dealing with type 1 diabetes, where the investigation of the role of C-peptide deficiency may play a significant role and is worth following up.

### IN VITRO INVESTIGATIONS

### Effect of C-peptide on eNOS

The vasodilator effect of insulin (Flynn et al., 1992; Tooke et al., 1985a) has previously been linked to the release of NO from endothelial cells, and a decreased endothelial NO production has been seen in diabetes (Forstermann et al., 1994; Moncada and Higgs, 1993). Therefore, we hypothesized that the vascular effects of C-peptide could also be related to the endothelial NO system. In accordance with the immediate onset of the vasodilator effects of C-peptide in vivo (Kunt et al., 1999), we initiated an in vitro study using a reporter cell assay in order to evaluate the possible effect of C-peptide on the activation of eNOS. We were able to demonstrate that C-peptide significantly enhanced the release of NO from bovine aortic endothelial cells (BAECs) (Figure 2). The effect of C-peptide in this study was dose dependent and the required doses for this stimulation have been in a physiological range of 1 to 6 nM.

These findings are in accordance to the results of Jensen and Messina (1999) who demonstrated a vasodilatory effect of C-peptide in isolated rat cremaster muscle arterioles, which was sensitive to $N^{G}$-nitro-L-arginine (L-NNA) inhibition, but in contrast to our results, the response of C-peptide was only observed in the presence of insulin.

Furthermore, it should be mentioned that in our study, the stimulation of NO was performed by stimulating BAECs with human C-peptide despite the fact that the whole C-peptide molecule varies largely in both species. Therefore, it is noteworthy that the C-terminal pentapeptide reveals apparently specific effects on $Na^{+}$,$K^{+}$-ATPase activity and shows partial homology between different species (Ohtomo et al., 1998). Moreover, recent studies of our group revealed that the C-terminal pentapeptide exerts similar effects compared to the whole peptide in this NO reporter cell assay and in the erythrocyte deformability assay as well, whereas non–C-terminal segments or a scrambled C-peptide showed no effect (Kunt et al., 1999; Hach et al., 2002).

C-peptide increased the intracellular $Ca^{2+}$ concentration in BAECs (Figure 3). Both the C-peptide–stimulated $Ca^{2+}$ signal and the NO release were abolished in $Ca^{2+}$-free medium because the endothelial eNOS is a $Ca^{2+}$-calmodulin–regulated enzyme (Forstermann et al., 1991), and the peptide is likely to stimulate eNOS activity by facilitating an influx of $Ca^{2+}$ into BAECs (Figure 3). C-peptide has previously been shown to increase $Ca^{2+}$ influx into renal tubular cells (Ohtomo et al., 1998), but similar data with endothelial cells have not been published as yet. In view of the important contribution of eNOS to the regulation of vascular resistance and blood pressure in vivo (Huang et al., 1995), a twofold increase in NO release is likely to have significant hemodynamic consequences.

Human endothelial cells in culture tend to loose their responsiveness to peptides very rapidly. Even human umbilical vein endothelial cells (HUVECs), which can be used at early passages, are much less reliable than BAECs in their response to receptor-mediated stimulation of NO release (Forstermann et al., 1989). Nevertheless, we repeated our experiments with HUVECs. As expected, the NO release from HUVECs in response to C-peptide (6.6 nM) was quite variable, with some

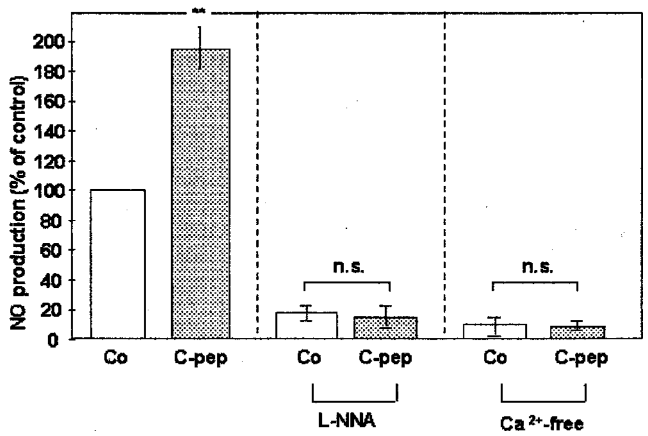

**FIGURE 2**
Effect of C-peptide on NO production in endothelial cells. There was a significant, almost 2-fold increase of NO production after incubation of bovine aortic endothelial cells with 6.6 nM C-peptide and evaluation in a reporter cell assay (RFL-6 cells). This activation of NO synthase could be blocked by addition of $N^{G}$-nitro-L-arginine (L-NNA) or in $Ca^{2+}$-free medium. 100% is equivalent to 5.01 ± 1.41 pmol cGMP/$10^{6}$ RFL-6 cells. Each column represents the mean ± SEM of 4 experiments (***$P < .001$ versus control).

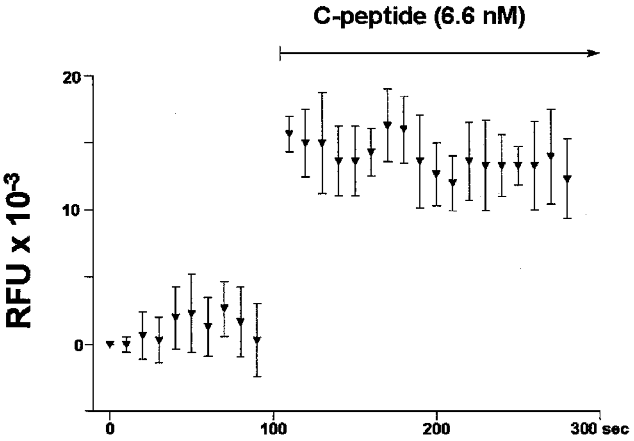

**FIGURE 3**
C-peptide–induced calcium influx into endothelial cells. Addition of C-peptide to bovine aortic endothelial cells yielded in a significant increase of calcium influx into the endothelial cells (measured by Fluo-3 technique).

batches of cells showing no significant NO production at all. Only freshly isolated cells showed measurable $Ca^{2+}$ influx and NO release in response to C-peptide. These data suggest that HUVECs in culture rapidly down-regulate or loose the binding site (receptor) for C-peptide. Also, Rigler and colleagues (1999) were unable to demonstrate specific membrane binding of C-peptide in cultures of HUVECs, but did show such binding in human saphenous vein endothelial cells, fibroblasts, and renal tubular epithelial cells.

The NO release from BAECs was smaller when the cells were exposed to the peptide for 30 minutes than after a 2-minute incubation. This transient effect suggests a desensitization of the potential receptor (or the subsequent signaling cascade), which has been also demostrated for other peptide stimulators of endothelial NO release (Forstermann et al., 1988). Moreover, endothelial cells have to be cultured at least 12 hours in serum-free medium in order to obtain a significant NO response (authors' own observations) because the potential C-peptide binding site seems to be saturated at low concentrations, which are certainly exceeded if the culture medium is supplemented with serum. Again, the study of Rigler and colleagues (1999) confirms this observation by demonstrating a competitive displacement of C-peptide at the binding site at very low concentrations.

In the same study, we were able to show by means of quantitative reverse transcriptase–polymerase chain reaction (RT-PCR) that the levels of eNOS mRNA are unlikely to be modulated by human C-peptide. This is in agreement with the immediate onset of the vascular effect of C-peptide, which could not have been explained by a change in the expression of eNOS.

In conclusion, C-peptide is able to stimulate an influx of $Ca^{2+}$ into endothelial cell, thereby activating the $Ca^{2+}$-sensitive eNOS and stimulating NO production. This may explain a major component of the vasodilator effect of human C-peptide and further strengthens the view that C-peptide indeed has biological effects.

### C-peptide and Red Cell Deformability

Diabetes mellitus is associated with morphological and functional alterations in microcirculation, heavily affecting hemorrheology. Beside increased capillary shunt flow due to peripheral diabetic neuropathy of the C fibers (Corbin et al., 1987), attenuated axon reflex response (Benarroch and Low, 1991) and increased leukocyte-endothelial interaction due to stimulated leukocyte integrins and endothelial adhesion molecules (Ruggiero et al., 1997; Kunt et al., 1998a), alteration of blood viscosity (Ernst and Matrai, 1986) is an important component of the hypothesis concerning the underlying mechanisms. Blood flow in larger vessels is determined by the vessel diameter, the viscosity (erythrocyte deformability and whole blood viscosity), and vessel length according to the law of Hagen-Pouiseuille. In smaller vessels of the microvasculature, especially if the diameter of the vessel is smaller than the diameter of the erythrocytes, as found in capillaries, blood flow is predominantly determined by the viscosity and deformability of the erythrocytes. Thus, reduced deformability leads to reduced flow in microcirculation if the capillary diameter and blood pressure remain constant (Chien, 1987).

The rheologic properties of erythrocytes in diabetes mellitus have been the subject of numerous studies. These studies demostrated that several factors, such as decreased erythrocyte deformability, increased erythrocyte aggregation, and increased membrane microviscosity, contribute to alterations of the rheological properties (Ernst and Matrai, 1986; Bareford et al., 1986; Finotti and Palatini, 1986; Chimori et al., 1986; Cohen et al., 1976; McMillan et al., 1998; Baba et al., 1979; Schmid-Schonbein and Volger, 1976) that are related to the modification of proteins and lipids by advanced glycation end products, to the generation of free oxygen radicals, and to changes in ion homeostasis by hyperglycemia.

Concerning the possible mechanism of reduced erythrocyte deformability, it is noteworthy that $Na^{+}$,$K^{+}$-ATPase activity has been shown to be attenuated in several cell types, including erythrocytes in diabetes patients (Ohtomo et al., 1996, 1998; Finotti and Palatini, 1986) and that it may be restored not only by insulin, but C-peptide as well (Ohtomo et al., 1996). This fact is considered to be of clinical importance because some type 1 diabetes patients maintain a measurable level of beta cell activity for many years, and the frequency of microvascular lesions in these patients is negatively correlated to residual islet cell function (Sjoberg et al., 1987; Kernell et al., 1990). These findings have generally been interpreted as indicating that remaining beta cell secretion of (endogenous) insulin exerts a beneficial effect on glycemic control. However, it is not apparent why endogenous insulin should be more protective than exogenous insulin.

These findings concerning the microcirculatory effects of C-peptide led to the suggestion that the peptide may also influence the rheologic properties of erythrocytes. Therefore, we investigated the deformability of erythrocytes in type 1 diabetes patients, i.e., C-peptide–deficient subjects, compared to healthy controls. Both groups were matched concerning their glucose levels in order to exclude a glucotoxic effect. Deformability was tested under physiological (0.3 to 10 Pa) and supraphysiological ($>$10 Pa) shear stress rates by means of laser diffractoscopy. The erythrocyte deformability was considerably lower in the diabetic group (Figure 4), e.g., at a physiological shear rate of 7.1 Pa, from $E = 0.4306 \pm 0.006$ to $E = 0.3794 \pm 0.023$ ($P = .002$).

During another study, erythrocytes from healthy controls and type 1 diabetes patients were incubated with different

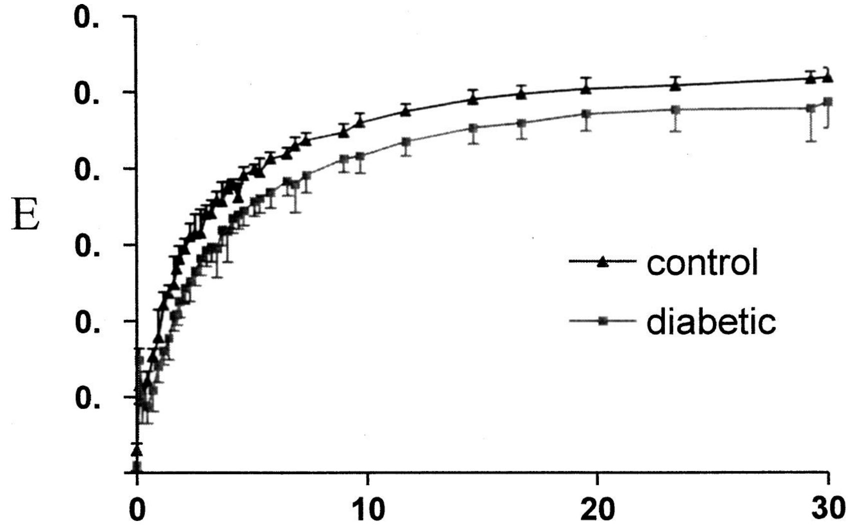

FIGURE 4
Measurement of elongation index E in diabetic patients and controls. Compared to healthy controls (▲), the elongation index E of the erythrocytes was considerably lower ($P < .0001$) in type 1 diabetes patients (■) at all tested shear rates. Statistical analysis was performed by 2-site ANOVA.

concentrations of C-peptide comparable to basal physiological (0.6 nM), postprandial physiological (6.6 nM), and supraphysiological (66.6 nM) levels. Interestingly, administration of C-peptide restored the formerly decreased deformability of diabetic erythrocytes compared to control erythrocytes of healthy volunteers, leaving a nonsignificant difference between the two groups (Figure 5). In contrast, proinsulin C-peptide had no additional effect on the deformability of erythrocytes in healthy controls. This might be explained by the fact that the control patients naturally revealed basal serum C-peptide levels, which were shown to exert already maximal effects on deformability in the present study. In agreement with these findings, the

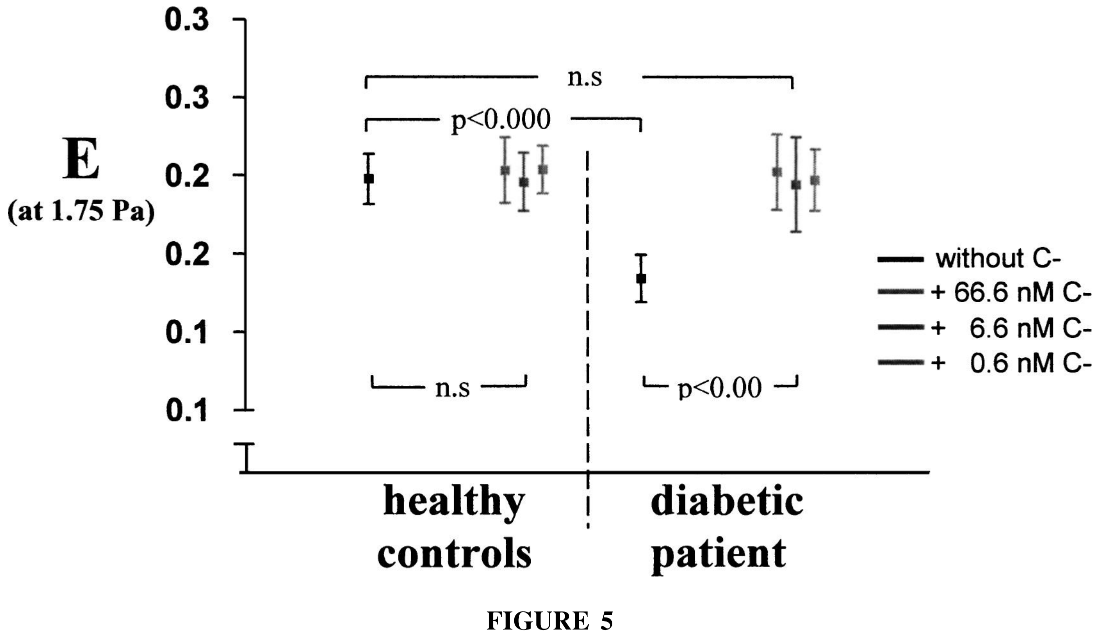

FIGURE 5
Representative analysis of erythrocyte deformability at 1.75 Pa. This graph shows the alterations of elongation index E at a shear stress of 1.75 Pa, which is frequently achieved in vivo. C-peptide did not modify the deformability of erythrocytes obtained from healthy controls, whereas the deformability of diabetic erythrocytes was restored to normal levels after administration of different concentrations of the peptide. Statistical analysis was performed by Student's $t$ test.

missing effect of proinsulin C-peptide on skin microcirculation in nondiabetic control subjects is worth mentioning (Forst et al., 1998).

Furthermore, different concentrations of C-peptide were found to exert similar effects on erythrocyte deformability. This finding underlines that physiological levels of proinsulin C-peptide are already sufficient to saturate C-peptide effects on erythrocyte deformability.

In order to hypothesize the potential mechanism of the effect of proinsulin C-peptide, previous studies concerning $Na^+$,$K^+$-ATPase activity in renal tubular cells and erythrocytes of diabetic subjects are of considerable interest (Ohtomo et al., 1996, 1998; Finotti and Palatini, 1986). Ohtomo and colleagues (1998, 1999) were able to show that the attenuated activitity of $Na^+$,$K^+$-ATPase activity in renal tubular segments of diabetic rats was able to be restored by proinsulin C-peptide. On the other hand, attenuation of $Na^+$,$K^+$-ATPase activity has been demonstrated to correlate with decreased deformability in erythrocytes of type 1 diabetes patients (Finotti and Palatini, 1986).

It is very speculative to explain the underlying mechanism based upon these results but impaired $Na^+$,$K^+$-ATPase activity may contribute to the decrease of erythrocyte deformability by increasing the intracellular sodium concentration, with subsequent intracellular accumulation of free calcium ions due to competition in transmembranous exchange (Mazzanti et al., 1990). These abnormalities in calcium homeostasis are known to enhance spectrin dimer-dimer interaction and spectrin–protein 4.1–actin interaction (Takakuwa and Mohandes, 1988; Schischmanoff et al., 1995). The latter is being promoted by adducin, a membrane skeleton–associated calmodulin-binding protein (Gardner and Bennett, 1986).

Hence, this effect of C-peptide on erythrocyte deformability is probably mediated by $Na^+$,$K^+$-ATPase restoration. Pretreatment of erythrocytes from type 1 diabetic patients with oubain, an $Na^+$,$K^+$-ATPase inhibitor, completely abolished C-peptide effects on erythrocyte deformability. Therefore it seems likely that the restoration of erythrocyte deformability by proinsulin C-peptide is mediated by an increase of $Na^+$,$K^+$-ATPase activity.

Recent studies with C-peptide fragments have shown that these effects of C-peptide on erythrocyte deformability are dependent on the presence of the C-terminal sequence of amino acids of the molecule (Hach et al., 2002).

### IN VIVO INVESTIGATIONS

### Effects of C-peptide on Microvascular Blood Flow

C-peptide has shown to influence microvascular blood flow by mediating vascular tonicity and increasing erythrocyte deformability. Therefore, it seems conceivable that C-peptide itself might exert biological effects on microvascular function and might have an implication in the development of microvascular complications in patients with type 1 diabetes mellitus.

In early in vitro studies, C-peptide had been shown to cause active vasodilatation of the microvasculature and to enhance recruitment of capillaries (Lindstrom et al., 1996). These results were confirmed in a study by Jensen and Messina (1999), which investigated the effect of C-peptide on skeletal muscle arterioles isolated from rat cremaster muscles. In this study, C-peptide evoked a concentration-independent arteriolar dilatation in a concentration range between 0.3 and 1000 ng/mL. This vasodilatation occurred within 10 minutes and reached its maximal effect within 15 minutes. Interestingly, addition of insulin at a concentration of 1 $\mu$U/mL, which had no vascular effect by its own, clearly enhanced the vascular effect of C-peptide. Therefore, it seems likely that insulin plays a permissive role in the vascular effects of C-peptide. Addition of L-NNA, an NOS inhibitor, completely abolished the vasodilating response of the microvessels, further indicating the role of NO in the mechanism of C-peptide action.

In a study done by Ido and colleagues (1997), beneficial effects of C-peptide supplementation could be documented in several vascular beds in diabetic rats. In their study, biosynthetic human C-peptide were given subcutaneously twice daily for 5 weeks in control rats and streptozotocin-induced diabetic rats. Highly supraphysiological peak plasma C-peptide levels between 9 and 10 nM were reached. C-peptide markedly attenuated the diabetes-induced increase in blood flow in the anterior uvea, retina, and sciatic nerve. In addition, C-peptide prevented an increased $^{125}$I-labeled albumin permeation in retina, nerve, and in the aorta. The improvement in microvascular blood flow was accompanied by an increase in caudal motor nerve conduction velocity. No effect of C-peptide, neither on microvascular blood flow nor on motor nerve conduction velocity, could be observed in the healthy control rats. Cotter and Cameron (2002) observed the vascular effects of C-peptide on sciatic endoneurial blood flow in streptozotocin diabetic rats at physiological C-peptide concentrations. In this study, C-peptide supplementation in streptozotocin-diabetic rats revealed an improvement in endoneurial blood flow and vascular conductance by 57% and 66% respectively. This improvement in endoneurial blood flow was accompanied by an improvement in motor nerve conduction velocity by 62% and sensory nerve conduction velocity by 78%. Cotreatment with L-NNA markedly attenuated the C-peptide effects on endoneurial blood flow and nerve conduction velocities.

Interestingly, at supraphysiological C-peptide levels reached in the study of Ido and colleagues (1997), a reverse sequence of C-peptide (retro-C-peptide) was almost as effective as native C-peptide with regard to the vascular effects. In contrast, a scrambled peptide in which the amino acid composition was identical to that of native C-peptide, but the sequence was randomized, was found to be ineffective. Intact human proinsulin and both split forms des(31,32) and des(64,65) were thought to be without any biological activity. Amino acid residues in the midportion of human C-peptide were found to be critical for the observed vascular functions of the peptide. From these findings, Ido and colleagues concluded that the effect of C-peptide might be mediated by a nonchiral mechanism rather than by stereospecific receptors on the cell membrane.

In an investigation by Johanssen and colleagues (1992), the effect of C-peptides on skeletal muscle blood flow were observed in type 1 diabetic patients and in healthy controls. In this study, forearm blood flow, capillary, diffusion capacity, and substrate exchange were studied during rhythmic forearm exercise on an hand ergometer. In the type 1 diabetic subjects, blood flow and capillary diffusion capacity of the exercising forearm were approximately 30% lower compared to the healthy control subjects. Intravenous administration of C-peptide during exercise increased forearm blood flow by 27% and capillary diffusion capacity by 52% to levels similar to those observed in the healthy controls. No significant changes in blood flow could be observed in the healthy controls receiving C-peptide or in the diabetic patients receiving placebo infusions.

In accordance with the observed improvements in skeletal muscle blood flow, forearm oxygen and glucose uptake increased markedly after C-peptide administration in type 1 diabetic patients. This study was the first to demonstrate that C-peptide administration has a significant hemodynamic and metabolic effect in human diabetic patients who lack endogeneous C-peptide secretion, whereas no such effect could be observed in healthy subjects.

As already mentioned before, C-peptide may act in synergism with other hormones. Neuropeptide Y (NPY) is a vasoconstrictor peptide that is costored with noradrenaline in sympathetic perivascular nerves. Type 1 diabetic patients have a blunted release of both noradrenaline and NPY during exercise (Ahlborg and Lundberg, 1996). Therefore, in a subsequent study, Johansson and Pernow (1999) examined whether there is an interference between NPY and C-peptide on forearm blood flow measured by venous occlusion plethysmography. During administration of C-peptide, forearm blood flow increased by 25%. The subsequent NPY infusion resulted in an enhanced vasconstrictor response of NPY. Forearm vascular resistance was inversely reduced during C-peptide administration (−17%) at baseline and during administration of NPY. Again, no effects of C-peptide, neither before nor during NPY administartion, could be observed in healthy controls.

Skin blood flow is found to be altered early after the diagnosis of diabetes mellitus (Tooke et al., 1985b; Ewald et al., 1981; Flynn and Tooke, 1992). The capillary circulation is functionally situated in parallel to the arteriovenous shunts and is thought to have the primary function of skin nutrition. It has been estimated that 80% to 90% of total skin blood flow passes through thermoregulatory arteriovenous shunts and does not enter the capillary bed (Jorneskog et al., 1995a, 1995b; Boulton et al., 1982). Although total skin perfusion is increased in diabetes mellitus, nutritional capillary skin blood flow was shown to be reduced in diabetic patients (Jorneskog et al., 1990, 1995a, 1995b). This discrepancy between nutritional and total skin microcirculation has been explained by a shunting of blood through arteriovenous anastomosis (Boulton et al., 1982; Flynn and Tooke, 1992). In one of our own investigations, we examined the effect of human C-peptide on skin microcirculation in type 1 diabetic patients and in healthy controls (Forst et al., 1998). In our study, total skin perfusion was measured by laserdopplerfluxmetry and nutritive capillary blood flow was investigated by intravital videophotometric capillaroscopy. At baseline, nutritive capillary blood flow was significantly lower in type 1 diabetic patients, compared with the control group. During C-peptide supplementation in type 1 diabetic patients, a steady increase in capillary blood flow could be observed, reaching values not different from the healthy control group after 60 minutes. No significant change in capillary blood flow could be observed in the healthy control group or in type 1 diabetic patients during placebo infusion. The capillary blood flow at baseline and after 60 minutes of infusion in the different groups are given in Figure 6. Thirty minutes after termination of C-peptide infusion, the capillary blood flow declined again to values not different from baseline.

A linear relationship was found between plasma C-peptide levels and the capillary blood flow velocity ($r = .401$, $P < .0001$). No impact of C-peptide could be observed on subpapillary blood flow measured by laserdopplerfluxmetry, neither in the type 1 diabetic group nor in the control group. In addition, spectral analysis of flow motion revealed no significant influence of C-peptide on neurovascular control.

The increase in plasma C-peptide levels was associated with an increase in nutritional capillary blood flow, however, did not affect total skin blood flow in insulin-dependent diabetes mellitus (IDDM) patients. These findings suggest a redistribution of skin microvascular blood flow into the nutritive capillary circulation during C-peptide administration. The increase in capillary blood flow, thus, may improve the exchange of nutrients, oxygen, and waste products in the skin. In healthy subjects, peak C-peptide levels are usually seen after the ingestion of food, with a complementary increase of glucose and nutrients in the blood stream. The postprandial

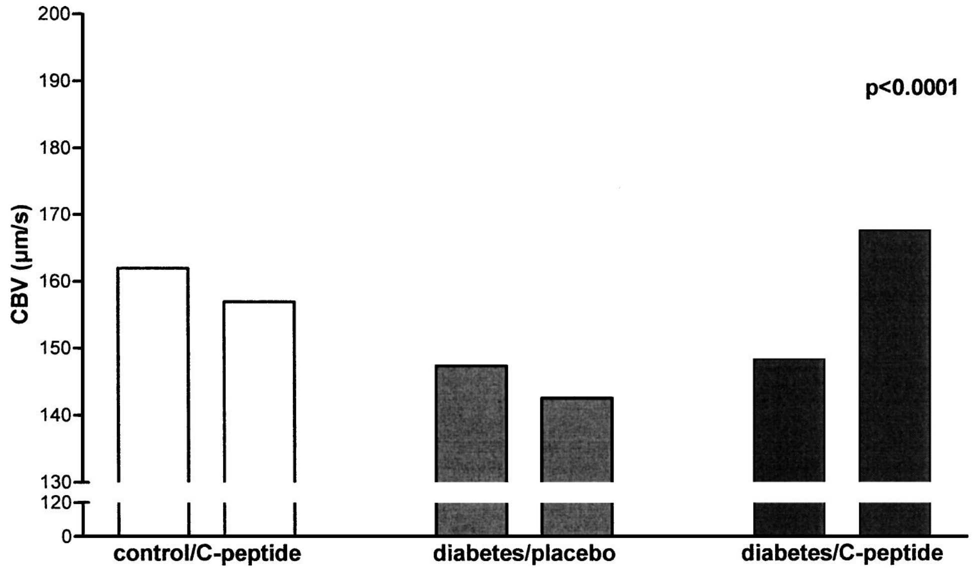

**FIGURE 6**
Nutritive capillary blood flow at baseline and 60 minutes after infusion of C-peptide (8 pmol/kg/min) in the different groups.

distribution of microvascular blood flow into nutritive vascular compartments may fulfill physiological requirements and the lack of endogenous C-peptide may lead to disturbances in nutritive tissue metabolism.

Also, insulin has been shown to increase skin capillary blood flow (Tooke et al., 1985a; Flynn et al., 1992) by modulation of endothelium-derived vasoactive substances, such as endothelin, NO, or adenosine (Poldermann et al., 1996). However, in our study, there was no significant difference in insulin levels between the different groups and observation periods and, therefore, the observed effects on microvascular blood flow may be attributed to the changes in C-peptide levels.

### Effects of C-peptide on Endothelial Cell Function and Endothelium–Blood Cell Interactions In Vivo

A couple of the previously described investigations gave evidence to the hypothesis that the vasodilatatory actions of C-peptide might be due to an effect on endothelial cells. The most widely known endothelium derived relaxing factor, NO, is released from endothelial cells in response to shear stress or stimulation of different receptors for a variety of neurohumoral mediators on the endothelial cell surface (Cohen and Vanhoutte, 1995). As described in the previous section, C-peptide has been shown to increase NO secretion in endothelial cells, which was clearly $Ca^{2+}$ dependent (Kunt et al., 1998b, 1998c).

Flow mediated vasodilatation (FMV) depends on an intact endothelial function and is considered to be mediated by NO (Palmer et al., 1987). In type 1 diabetes, FMV is known to be decreased in conduit arteries, such as the brachial or femoral arteries (Elliott et al., 1993; Johnstone et al., 1993; Clarkson et al., 1996).

Fernqvist-Forbes and colleagues (2001) studied the effect of C-peptide administration on flow-mediated vasodilatation (FMV) in response to reactive hyperaemia in type 1 diabetic patients. In addition, the arterial dilatation to glyceryl trinitrate, which is an endothelium-independent marker of vascular smooth muscle function, was investigated. When compared with a healthy control group, the type 1 diabetic patients revealed a lower flow-mediated dilatation. Following C-peptide administration, basal blood flow in the brachial artery increased by approximately 35% compared with the control measurement when NaCl was infused. In response to reactive hyperemia, C-peptide administration resulted in a significant increase in forearm blood flow compared with the basal state and with the control day. During saline infusion, no significant changes in forearm blood flow could be observed. No significant changes after glyceryltrinitrate, neither during C-peptide infusion nor during saline infusion, could be obtained. In addition, a slight, but significant, increase in the left ventricular systolic function could be found in type 1 diabetic patients during C-peptide infusion, whereas no effect was observed during NaCl infusion. In conclusion, this study underlines an endothelium-dependent mechanism in C-peptide action. No effect of C-peptide could be observed on endothelium-independent blood flow regulation.

In one of our own studies, we explored the effect of C-peptide on acetylcholine-evoked vasodilatation in skin microcirculation in type 1 diabetic patients (Forst et al., 2000). Acetylcholine elicts vasodilatation through a stimulation of eNOS, with an

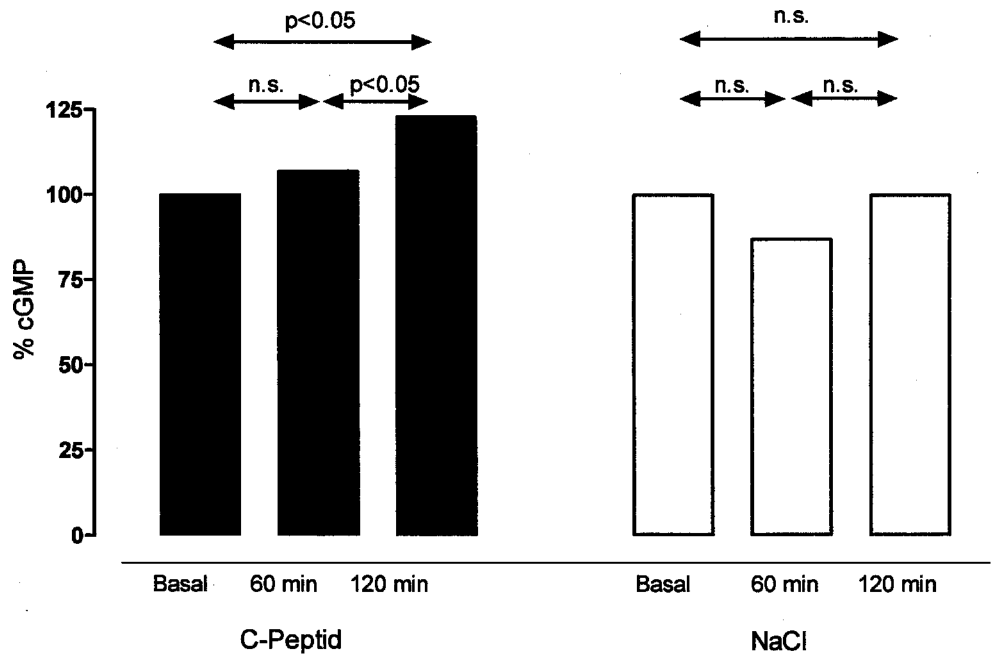

**FIGURE 7**
Cyclic guanyl monophosphate (cGMP) at baseline and after 60 minutes (3 pmol/kg/min) and 120 minutes (10 pmol/kg/min) of C-peptide or placebo infusion.

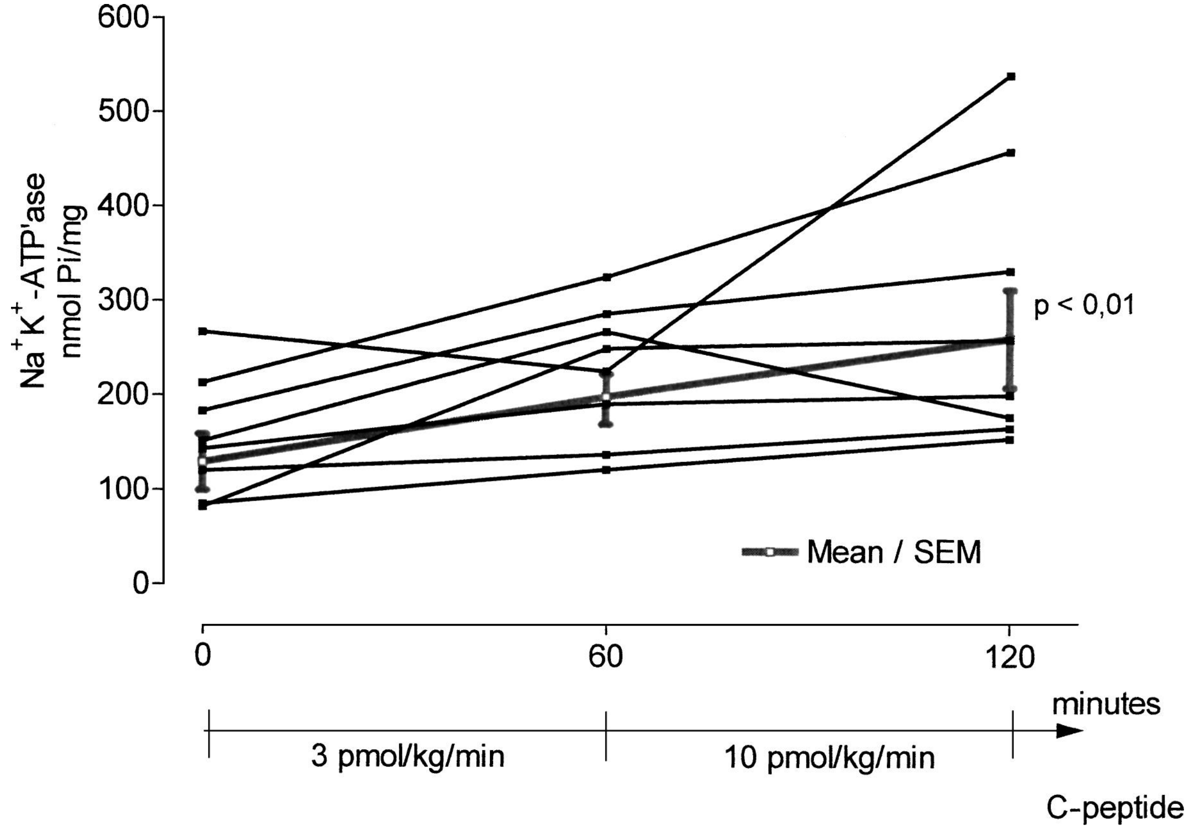

**FIGURE 8**
$Na^+$,$K^+$-ATPase activity at baseline and after 60 minutes (3 pmol/kg/min) and 120 minutes (10 pmol/kg/min) of C-peptide infusion.

increased NO production and a subsequent increase of cyclic GMP in vascular smooth muscle (Calles-Escandon and Cipolla, 2001; Morris et al., 1995; Pieper et al., 1997; McNally et al., 1994). In this study, the skin microvascular response was measured by laser doppler fluxmetry and acetylcholine was applied to the dorsum of the foot by the technique of iontophoresis. C-peptide was infused intravenously at a concentration of 3 $pmol^{-1}$ $kg^{-1}$ $min^{-1}$ for the first 60 minutes and at a concentration of 10 pmol $kg^{-1}$ $min^{-1}$ for another 60 minutes. At baseline and after 60 and 120 minutes, the laser doppler flux response to acetylcholine was measured and blood samples were taken for the measurement of plasma cyclic GMP levels. The laserdopplerflux response to acetylcholine increased by 133% during the low infusion rate and by 80% during the high infusion rate of C-peptide. The increase in the laser doppler flux response to acetylcholine was significant compared to baseline and to a saline infusion. No significant difference in the laserdopplerflux response to acetylcholine could be observed between the different C-peptide concentrations. Cyclic GMP levels are illustrated in Figure 7.

There was found a 14% increase in plasma cyclic GMP levels during the low infusion rate of C-peptide and a 23% increase in plasma cyclic GMP levels during the high infusion rate of C-peptide ($P < .05$). No significant changes in plasma C-peptide levels could be observed during NaCl infusion.

### Effects of C-peptide on Erythrocyte $NA^+$,$K^+$-ATPase Activity

In a recent investigation by Dufayet and colleagues (1998), erythrocyte $Na^+$,$K^+$-ATPase activity was compared between type 1 and type 2 diabetic patients. Although in type 1 diabetic patients, a clear reduction in erythrocyte $Na^+$,$K^+$-ATPase activity could be observed, type 2 diabetic patients exhibited a wide range of individual $Na^+$,$K^+$-ATPase activity readings, presenting some patients with very low $Na^+$,$K^+$-ATPase activity and others with $Na^+$,$K^+$-ATPase activity within the physiological range. It appeared that erythrocyte $Na^+$,$K^+$-ATPase activity was significantly lower in those type 2 patients treated with insulin compared with those on oral treatment. Also in the former, $Na^+$,$K^+$-ATPase activity was comparable to those in type 1 diabetic patients. The authors suggested that treatment with exogenous insulin in type 2 diabetic patients could negatively regulate $Na^+$,$K^+$-ATPase activity. Type 2 diabetic patients were older, had a longer diabetes duration, poorer glycemic control, and lower C-peptide levels, but only C-peptide was independently correlated with

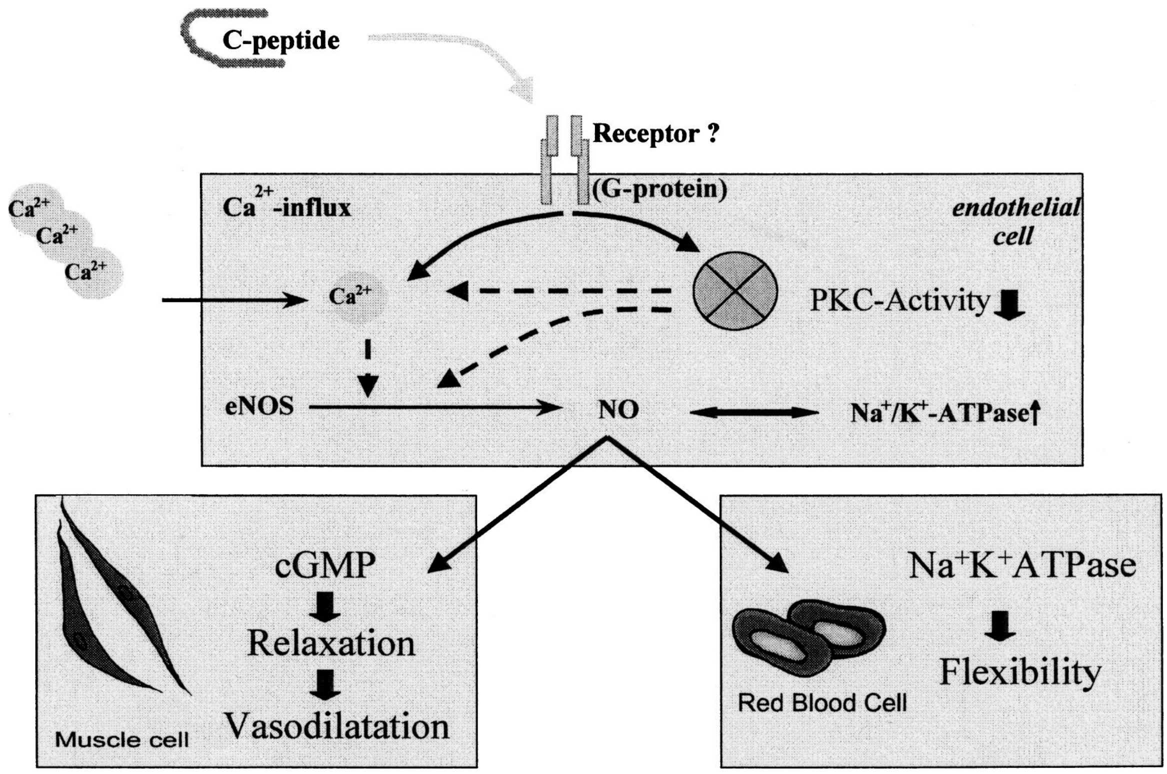

**FIGURE 9**
Postulated molecular mechanism of C-peptides vascular and hemorheological effects.

erythrocyte $Na^+$,$K^+$-ATPase activity. In an in vitro study by Djemli-Shiplolye and colleagues (2000), incubation of erythrocytes from type 1 diabetic patients with C-peptide showed a significant increase in erythrocyte $Na^+$,$K^+$-ATPase activity. In one of our own studies, intravenous infusion of C-peptide in a concentration of 3 pmol/kg/min for 60 minutes and at a concentration of 10 pmol/kg/min for additional 60 minutes in type 1 diabetic patients resulted in a steady increase in erythrocyte $Na^+$,$K^+$-ATPase activity (Figure 8), whereas infusion of NaCl was without any significant effect (Forst et al., 2000).

### CONCLUSIONS

The potential role of C-peptide as an biologically active peptide has been controversially discussed in the past on the basis of studies being contradictory concerning the impact of this cleavage product of insulin. Recent studies, in turn, demonstrated unanimously that C-peptide is biologically active by predominantly modulating blood flow. Thus, there is evidence that C-peptide improves red cell deformability and microvascular blood flow in type 1 diabetes. The underlying mechanisms, initiated by binding to an G-protein–coupled receptor, involve at least the activation of eNOS and the activation of $Na^+$,$K^+$-ATPase and are therefore calcium dependent and ouabain sensitive. The postulated mechanism by which C-peptide interact with microvascular blood flow is illustrated in Figure 9.

Therefore, it is fairly reasonable to advance the hypothesis that C-peptide supplementation in a state of C-peptide deficiency (e.g., in type 1 diabetes) may be beneficial in order to prevent nonglucotoxic vascular complications. The improvement of the formerly impaired hemorrheological properties of vascular regulation as demonstrated by these investigations need certainly further support by well-designed prospective interventional clinical trials monitoring the progression of vascular complications in a long-term follow-up.

### REFERENCES

Ahlborg, G., and Lundberg, J. M. (1996) Exercise-induced changes in neuropeptide Y, noradrenaline and endothelin-1 levels in young people with type I diabetes. *Clin. Physiol.,* **16,** 645–655.

Baba, Y., Kai, M., Kamada, T., Setoyama, S., and Otsuji, S. (1979) Higher levels of erythrocyte membrane microviscosity in diabetes. *Diabetes,* **28,** 1138–1140.

Bareford, D., Jennings, P. E., Stone, P. C., Baar, S., Barnett, A. H., and Stuart, J. (1986) Effects of hyperglycaemia and sorbitol accumulation on erythrocyte deformability in diabetes mellitus. *J. Clin. Pathol.,* **39,** 722–727.

Barnes, A. J., Locke, O., Scudder, P. R., Dormandy, T. L., and Slack, J. (1977) Is hyperviscosity a treatable component of diabetic microcirculatory disease. *Lancet,* **2,** 789–791.

Benarroch, E. E., and Low, P. A. (1991) The acetylcholine-induced flare response in evaluation of small fiber dysfunction. *Ann. Neurol.,* **29,** 590–595.

Boulton, A. J., Scarpello, J. H. B., and Ward, J. D. (1982) Venous oxygenation in the diabetic neuropathic foot: Evidence of arteriovenous shunting. *Diabetologia,* **22,** 6–8.

Calles-Escandon, J., and Cipolla, M. (2001) Diabetes and endothelial dysfunction: A clinical perspective. *Endocr. Rev.,* **22,** 36–52.

Chien, S. (1987) Red cell deformability and its relevance to blood flow. *Annu. Rev. Physiol.,* **49,** 177–192.

Chimori, K., Miyazaki, S., Kosaka, J., Sakanaka, A., Yasuda, K., and Miura, K. (1986) Increased sodium influx into erythrocytes in diabetes mellitus and hypertension. *Clin. Exp. Hypertens A,* **8,** 185–199.

Clarkson, P., Celermajer, D. S., Donald, A. E., Sampson, M., Sorensen, K. E., Adams, M., Yue, D. K., Betteridge, D. J., and Deanfield, J. E. (1996) Impaired vascular reactivity in insulin-dependent diabetes mellitus is related to disease duration and low density lipoprotein cholesterol levels. *J. Am. Coll. Cardiol.,* **28,** 573–579.

Cohen, N. S., Ekholm, J. E., Luthra, M. G., and Hanahan, D. J. (1976) Biochemical characterization of density-separated human erythrocytes. *Biochim. Biophys. Acta,* **419,** 229–242.

Cohen, R. A., and Vanhoutte, P. M. (1995) Endothelium-dependent hyperpolarization. Beyond nitric oxide and cyclic GMP. *Circulation,* **92,** 3337–3349.

Colwell, J. A., and Lopes-Virella, M. F. (1988) A review of the development of large-vessel disease in diabetes mellitus. *Am. J. Med.,* **85,** 113–118.

Corbin, D. O. C., Young, R. J., Morrison, D. C., Hoskins, P., McDicken, W. N., Housley, E., Clarke, B. F. (1987) Blood flow in the foot, polyneuropathy and foot ulceration in diabetes mellitus. *Diabetologia,* **30,** 468–473.

Cotter, M. A., and Cameron, N. E. (2001) C-peptide effects on nerve conduction and blood flow in streptozotocin-induced diabetic rats: Modulation by nitric oxide synthase inhibition. *Diabetes,* **51**(Suppl. 2), A184.

Danthuluri, N. R., Cybulsky, M. I., and Brock, T. A. (1988) ACh-induced calcium transients in primary cultures of rabbit aortic endothelial cells. *Am. J. Physiol.,* **255,** H1549–H1553.

Djemli-Shipkolye, A., Gallice, P., Coste, T., Jannot, M. F., Tsimaratos, M., Raccah, D., and Vague, P. (2000) The effects ex vivo and in vitro of insulin and C-peptide on Na/K adenosine triphosphatase activity in red blood cell membranes of type 1 diabetic patients. *Metabolism,* **49,** 868–872.

Dufayet, D., Raccah, D., Jannot, M. F., Coste, T., Rougerie, C., and Vague, P. (1998) Erythrocyte Na/K ATPase activity and diabetes: Relationship with C-peptide level. *Diabetologia,* **41,** 1080–1084.

Elliott, T. G., Cockcroft, J. R., Groop, P. H., Viberti, G. C., and Ritter, J. M. (1993) Inhibition of nitric oxide synthesis in forearm vasculature of insulin-dependent diabetic patients: Blunted vasoconstriction in patients with microalbuminuria. *Clin. Sci. (Lond.),* **85,** 687–693.

Ernst, E., and Matrai, A. (1986) Altered red and white blood cell rheology in type II diabetes. *Diabetes,* **35,** 1412–1415.

Ewald, U., Tuvemo, T., and Rooth, G. (1981) Early reduction of vascular reactivity in diabetic children detected by transcutaneous oxygen electrode. *Lancet,* **1,** 1287–1288.

Fernqvist-Forbes, E., Johansson, B. L., and Eriksson, M. J. (2001) Effects of C-peptide on forearm blood flow and brachial artery dilatation in patients with type 1 diabetes mellitus. *Acta. Physiol. Scand.,* **172,** 159–165.

Finotti, P., and Palatini, P. (1986) Reduction of erythrocyte ($Na^+K^+$)ATPase activity in type 1 (insulin-dependent) diabetic subjects. *Diabetologia,* **29,** 623–628.

Flynn, M. D., Boolell, M., Tooke, J. E, and Watkins, P. J. (1992) The effect of insulin infusion on capillary blood flow in the diabetic neuropathic foot. *Diabet. Med.,* **9,** 630–634.

Flynn, M. D., and Tooke, J. E. (1992) Aetilogy of diabetic foot ulceration. *Diabetic Med.,* **8,** 320–329.

Forst, T., De La Tour, D. D., Kunt, T., Pfutzner, A., Goitom, K., Pohlmann, T., Schneider, S., Johansson, B. L., Wahren, J., Lobig, M., Engelbach, M., Beyer, J., and Vague, P. (2000) Effects of proinsulin C-peptide on nitric oxide, microvascular blood flow and erythrocyte $Na^+$,$K^+$-ATPase activity in diabetes mellitus type I. *Clin. Sci.,* **98,** 283–290.

Forst, T., Kunt, T., Pohlmann, T., Goitom, K., Engelbach, M., Beyer, J., and Pfutzner, A. (1998) Biological activity of C-peptide on the skin microcirculation in patients with insulin-dependent diabetes mellitus. *J. Clin. Invest.,* **101,** 2036–2041.

Forst, T., Pfutzner, A., Bauersachs, R., Arin, M., Bach, B., Biehlmaier, H., Kustner, E., and Beyer, J. (1997) Comparison of the microvascular response to transcutaneous electrical nerve stimulation and postocclusive ischemia in the diabetic foot. *J. Diabetes Complications,* **11,** 291–297.

Forstermann, U., Closs, E. I., Pollock, J. S., Nakane, M., Schwarz, P., Gath, I., and Kleinert, H. (1994) Nitric oxide synthase isozymes. Characterization, purification, molecular cloning, and functions. *Hypertension,* **23,** 1121–1131.

Forstermann, U., Mugge, A., Alheid, U., Haverich, A., and Frolich, J. C. (1988) Selective attenuation of endothelium-mediated vasodilation in atherosclerotic human coronary arteries. *Circ. Res.,* **62,** 185–190.

Forstermann, U., Pollock, J. S., Schmidt, H. H., Heller, M., and Murad, F. (1991) Calmodulin-dependent endothelium-derived relaxing factor/nitric oxide synthase activity is present in the particulate and cytosolic fractions of bovine aortic endothelial cells. *Proc. Natl. Acad. Sci. U. S. A.,* **88,** 1788–1792.

Forstermann, U., Warmuth, G., Dudel, C., and Alheid, U. (1989) Formation and functional importance of endothelium-derived relaxing factor (EDRF) and prostaglandins in the microcirculation. *Z. Kardiol.,* **78** (Suppl 6), 85–91.

Furchgott, R. F., and Zawadzki, J. V. (1980) The obligatory role of endothelial cells in the relaxation of arterial smooth muscle by acetylcholine. *Nature,* **288,** 373–376.

Gardner, K., and Bennett, V. (1986) A new erythrocyte membrane-associated protein with calmodulin binding activity. Identification and purification. *J. Biol. Chem.,* **261,** 1339–1348.

Gawler, D., Milligan, G., Spiegel, A. M., Unson, C. G., and Houslay, M. D. (1987) Abolition of the expression of inhibitory guanine nucleotide regulatory protein Gi activity in diabetes. *Nature,* **327,** 229–232.

Gerbi, A., Barbey, O., Raccah, D., Coste, T., Jamme, I., Nouvelot, L., Ouafik, L., Levy, S., Vague, P., and Maixent, J. M. (1997) Alteration of Na,K-ATPase isoenzymes in diabetic cardiomyopathy: Effect of dietary supplementation with fish oil (n-3 fatty acids) in rats. *Diabetologia,* **40,** 496–505.

Greene, D. A., Lattimer, S. A., and Sima, A. A. (1987) Sorbitol, phosphoinositides, and sodium-potassium-ATPase in the pathogenesis of diabetic complications. *N. Engl. J. Med.,* **316,** 599–606.

Gupta, S., McArthur, C., Grady, C., and Ruderman, N. B. (1994) Stimulation of vascular Na(+)-K(+)-ATPase activity by nitric oxide: A cGMP-independent effect. *Am. J. Physiol.,* **266,** H2146–H2151.

Hach, T., Johansson, B. L., Ekberg, K., Forst, T., Kunt, T., and Wahren, J. (2002) C-peptide and its fragments increase red blood cell deformability in type 1 diabetes. *Diabetes,* **51,** A621.

Huang, P. L., Huang, Z., Mashimo, H., Bloch, K. D., Moskowitz, M. A., Bevan, J. A., and Fishman, M. C. (1995) Hypertension in mice lacking the gene for endothelial nitric oxide synthase. *Nature,* **377,** 239–242.

Ido, Y., Vindigni, A., Chang, K., Stramm, L., Chance, R., Heath, W. F., DiMarchi, R. D., Di Cera, E., and Williamson, J. R. (1997) Prevention of vascular and neural dysfunction in diabetic rats by C-peptide. *Science,* **277,** 563–566.

Jensen, M. E., and Messina, E. J. (1999) C-peptide induces a concentration-dependent dilation of skeletal muscle arterioles only in presence of insulin. *Am. J. Physiol.,* **276,** H1223–H1228.

Johansson, B. L., Linde, B., and Wahren, J. (1992) Effects of C-peptide on blood flow, capillary diffusion capacity and glucose utilization in the exercising forearm of type 1 (insulin-dependent) diabetic patients. *Diabetologia,* **35,** 1151–1158.

Johansson, B. L., and Pernow, J. (1999) C-peptide potentiates the vasoconstrictor effect of neuropeptide Y in insulin-dependent diabetic patients. *Acta Physiol. Scand.,* **165,** 39–44.

Johnstone, M. T., Craeger, S. J., Scales, K. M., Cusco, J. A., Lee, B. A., and Craeger, M. A. (1993) Impaired endothelium-dependent vasodilatation in patients with insulin-dependent diabetes mellitus. *Circulation,* **88,** 2510–2516.

Jorneskog, G., Brismar, K., and Fagrell, B. (1995a) Skin capillary circulation is more impaired in the toes of diabetic than non-diabetic patients with peripheral vascular disease. *Diabet. Med.,* **12,** 36–41.

Jorneskog, G., Brismar, K., and Fagrell, B. (1995b) Skin capillary circulation severly impaired in toes of patients with IDDM, with and withourt late diabetic complications. *Diabetologia,* **38,** 474–480.

Jorneskog, G., Ostergren, J., Tyden, G., Bolinder, J., and Fagrell, B. (1990) Does combined kidney and pancreas transplantation reverse functional diabetic microangiopathy? *Transplant. Int.,* **3,** 167–170.

Kernell, A., Ludvigsson, J., and Finnström, K. (1990) Vitreous fluorophotometry in juvenile diabetics with and without retinopathy in relation to metabolic control: Insulin antibodies and C-peptide levels. *Acta Ophthalmol. Scand.,* **68,** 415–420.

Kunt, T., Forst, T., Harzer, O., Buchert, G., Pfutzner, A., Lobig, M., Zschabitz, A., Stofft, E., Engelbach, M., and Beyer, J. (1998a) The influence of advanced glycation endproducts (AGE) on the expression of human endothelial adhesion molecules. *Exp. Clin. Endocrinol. Diabetes,* **106,** 183–188.

Kunt, T., Forst, T., Lehmann, R., Pfutzner, A., Löbig, M., Harzer, O., Engelbach, M., and Beyer, J. (1998b) Human C-peptide increases calcium influx into endothelial cells. *Diabetes,* **47** (Suppl 1), A30.

Kunt, T., Forst, T., Pfutzner, A., Beyer, J., and Wahren, J. (1998c) The physiological impact of proinsulin C-peptide. *Pathophysiology,* **5,** 257–262.

Kunt, T., Schneider, S., Pfutzner, A., Goitom, K., Engelbach, M., Schauf, B., Beyer, J., and Forst, T. (1999) The effect of human proinsulin C-peptide on erythrocyte deformability in patients with type 1 diabetes mellitus. *Diabetologia,* **42,** 465–471.

Lee, T. S., MacGregor, L. C., Fluharty, S. J., and King, G. L. (1989) Differential regulation of protein kinase C and (Na,K)-adenosine triphosphatase activities by elevated glucose levels in retinal capillary endothelial cells. *J. Clin. Invest.,* **83,** 90–94.

Lindstrom, K., Johansson, C., Johnsson, E., and Haraldsson, B. (1996) Acute effects of C-peptide on the microvasculature of isolated perfused skeletal muscles and kidneys in rat. *Acta Physiol. Scand.,* **156,** 19–25.

Mazzanti, L., Rabini, R. A., Faloia, E., Fumelli, P., Bertoli, E., and DePirro, R. (1990) Altered cellular $Ca^{2+}$ and $Na^{+}$ transport in diabetes mellitus. *Diabetes,* **39,** 850–854.

McMillan, D. E., Utterback, N. G., and LaPuma, J. (1998) Reduced erythrocyte deformability in diabetes. *Diabetes,* **27,** 895–901.

McNally, P. G., Watt, P. A. C., Rimmer, T., Burden, A. C., Hearnshaw, J. R., and Thurston, H. (1994) Impaired contraction and endothelium-dependent relaxation in isolated resistance vessels from patients with insulin-dependent diabetes mellitus. *Clin. Sci.,* **87,** 31–36.

Moncada, S., and Higgs, A. (1993) The L-arginine-nitric oxide pathway. *N. Engl. J. Med.,* **329,** 2002–2012.

Morris, S. J., Shore, A. C., and Tooke, J. E. (1995) Responses of the skin microcirculation to acetylcholine and sodium nitroprusside in patients with NIDDM. *Diabetologia,* **38,** 1337–1344.

Netten, P. M., Wollersheim, H., Thien, T., and Lutterman, J. A. (1996) Skin microcirculation of the foot in diabetic neuropathy. *Clin. Sci.,* **91,** 559–565.

Ohtomo, Y., Aperia, A., Sahlgren, B., Johansson, B. L., and Wahren, J. (1996) C-peptide stimulates rat renal tubular Na+, K(+)-ATPase activity in synergism with neuropeptide Y. *Diabetologia,* **39,** 199–205.

Ohtomo, Y., Bergman, T., Johansson, B. L., Jornvall, H., and Wahren, J. (1998) Differential effects of proinsulin C-peptide fragments on $Na^{+}$, $K^{+}$-ATPase activity of renal tubule segments. *Diabetologia,* **41,** 287–291.

Palmer, R. M., Ferrige, A. G., and Moncada, S. (1987) Nitric oxide release accounts for the biological activity of endothelium-derived relaxing factor. *Nature,* **327,** 524–526.

Peach, M. J., Singer, H. A., Izzo, N. J., Jr., and Loeb, A. L. (1987) Role of calcium in endothelium-dependent relaxation of arterial smooth muscle. *Am. J. Cardiol.,* **59,** 35A–43A.

Pieper, G. M., Siebeneich, W., Moore-Hilton, G., and Roza, A. M. (1997) Reversal by L-arginine of a dysfunctional arginine/nitric oxide pathway in the endothelium of the genetic diabetic BB rat. *Diabetologia,* **40,** 910–915.

Poldermann, K. H., Stehouwer, C. D. A., vanKamp, G. J., and Gooren, L. J. G. (1996) Effects of insulin infusion on endothelium-derived vasoactive substances. *Diabetologia,* **39,** 1284–1292.

Rand, V. E., and Garland, C. J. (1992) Endothelium-dependent relaxation to acetylcholine in the rabit basilar artery: Importance of membrane hyperpolarization. *Br. J. Pharmacol.,* **106,** 143–150.

Rapoport, R. M., Schwartz, K., and Murad, F. (1985) Effects of $Na^{+}K^{+}$-pump inhibitors and membrane depolarizing agents on acetycholine-induced endothelium dependent relaxation and cyclic GMP accumulation in rat aorta. *Eur. J. Pharmacol.,* **110,** 203–209.

Rayman, G., Malik, R. A., Sharma, A. K., and Day, J. L. (1995) Microvascular response to tissue injury and capillary ultrastructure in the foot skin of type I diabetic patients. *Clin. Sci.,* **89,** 467–474.

Rigler, R., Pramanik, A., Jonasson, P., Kratz, G., Jansson, O. T., Nygren, P., Stahl, S., Ekberg, K., Johansson, B., Uhlen, S., Uhlen, M., Jornvall, H., and Wahren, J. (1999) Specific binding of proinsulin C-peptide to human cell membranes. *Proc. Natl. Acad. Sci. U. S. A.,* **96,** 13318–13323.

Rose, A. M., and Valdes, R., Jr. (1994) Understanding the sodium pump and its relevance to disease. *Clin. Chem.,* **40,** 1674–1685.

Ruggiero, D., Lecomte, M., Michoud, E., Lagarde, M., and Wiernsperger, N. (1997) Involvement of cell-cell interactions in the pathogenesis of diabetic retinopathy. *Diabete Metab.,* **23,** 30–42.

Sandemann, D. D., Shore, A. C., and Tooke, J. E. (1992) Relation of skin capillary pressure in patients with insulin-dependent diabetes mellitus to complications and metabolic control. *N. Engl. J. Med.,* **327,** 760–764.

Schischmanoff, P. O., Winardi, R., Discher, D. E., Parra, M. K., Bicknese, S. E., Witkowska, H. E., Conboy, J. G., and Mohandas, N. (1995) Defining of the minimal domain of protein 4.1 involved in spectrin-actin binding. *J. Biol. Chem.,* **270,** 21243–21250.

Schmid-Schonbein, H., and Volger, E. (1976) Red-cell aggregation and red-cell deformability in diabetes. *Diabetes,* **25,** 897–902.

Simmons, D. A., Kern, E. F., Winegrad, A. I., and Martin, D. B. (1986) Basal phosphatidylinositol turnover controls aortic $Na^{+}/K^{+}$-ATPase activity. *J Clin. Invest.,* **77,** 503–513.

Sjoberg, S., Gunnarsson, R., Gjotterberg, M., Lefvert, A. K., Persson, A., and Ostman, J. (1987) Residual insulin production, glycaemic control and prevalence of microvascular lesions and polyneuropathy in long-term type 1 (insulin-dependent) diabetes mellitus. *Diabetologia,* **30,** 208–213.

Tack, C. J., Lutterman, J. A., Vervoort, G., Thien, T., and Smits, P. (1996) Activation of the sodium-potassium pump contributes to insulin-induced vasodilation in humans. *Hypertension,* **28,** 426–432.

Takakuwa, Y., and Mohandes, N. (1988) Modulation of erythrocyte membrane material properties by $Ca^{2+}$ and calmodulin. *J. Clin. Invest.,* **82,** 394–400.

Tesfamariam, B., Jakubowski, J. A., and Cohen, R. A. (1989) Contraction of diabetic rabbit aorta caused by endothelium-derived PGH2-TxA2. *Am. J. Physiol.,* **257,** H1327–H1333.

Tooke, J. E. (1999) Microvascular function in human diabetes. A physiological perspective. *Diabetes,* **44,** 721–726.

Tooke, J. E., Lins, P. E., Ostergren, J., Adamson, U., and Fagrell, B. (1985a) The effects of intravenous insulin infusion on skin microcirculatory flow in type 1 diabetes. *Int. J. Microcirc. Clin. Exp.,* **4,** 69–83.

Tooke, J. E., Lins, P. E., Ostergren, J., and Fagrell, B. (1985b) Skin microvascular autoregulatory responses in type I diabetes: The influence of duration and control. *Int. J. Microcirc. Clin. Exp.,* **4,** 249–256.

Vague, P., Dufayet, D., Coste, T., Moriscot, C., Jannot, M. F., and Raccah, D. (1997) Association of diabetic neuropathy with Na/K ATPase gene polymorphism. *Diabetologia,* **40,** 506–511.

Wald, H. P., Scherzer, P., Rasch, R., and Popovtzer, M. M. (1993) Renal tubular Na,K-ATPase in diabetes mellitus: Relationship to metabolic abnormality. *Am. J. Physiol.,* E96–E101.

